# Hepatocellular carcinoma among US and non-US-born patients with chronic hepatitis B: Risk factors and age at diagnosis

**DOI:** 10.1371/journal.pone.0204031

**Published:** 2018-09-25

**Authors:** Kaitlyn Kennedy, Susan M. Graham, Nayan Arora, Margaret C. Shuhart, H. Nina Kim

**Affiliations:** 1 Department of Global Health, School of Medicine, University of Washington, Seattle, Washington, United States of America; 2 Department of Medicine, Division of Allergy & Infectious Diseases, University of Washington, Seattle, Washington, United States of America; 3 Department of Medicine, Divison of Nephrology, University of Washington, Seattle, Washington, United States of America; 4 Department of Medicine, Division of Gastroenterology, University of Washington, Seattle, Washington, United States of America; Centre de Recherche en Cancerologie de Lyon, FRANCE

## Abstract

**Background:**

Risk factors for hepatocellular carcinoma (HCC) have not been well characterized among African immigrants with chronic hepatitis B virus (HBV) infection. We conducted a case-control study to identify demographic and clinical factors associated with HCC among a diverse cohort of patients with chronic HBV infection seen in a large academic health setting.

**Methods:**

We identified a total of 278 patients with HCC and chronic HBV seen at two medical centers in a 14-year span from January 2002 to December 2015. These cases were age- and sex-matched in a 1:3 ratio with 823 non-cancer control subjects with chronic HBV. Conditional logistic regression was used to estimate the odds of HCC by race, with black race stratified by African-born status, after adjusting for diabetes, HIV or HCV coinfection, alcohol misuse and cirrhosis.

**Results:**

Of the 278 HCC cases, 67% were 60 years of age or older, 78% were male, 87% had cirrhosis and 72% were Asian. HIV infection was present in 6% of cases. Only 7% (19 of 278) of HCC cases were black, of whom 14 were African immigrants. The median age at HCC diagnosis was 44 years in Africans. Notably, nearly all (93%) of the African-born patients with HCC were diagnosed at an age younger than 60 years compared with 52% of Asian cases (*P*<0.001). The main factors independently associated with greater odds of HCC overall were Asian race (adjusted odds ratio [aOR] 3.3, 95% confidence interval [CI] 1.9–5.5) and cirrhosis (aOR 19.7, 95% CI 12.2–31.8).

**Conclusion:**

African immigrants accounted for a small proportion of HBV-associated HCC cases overall compared with Asians but appeared to have greater likelihood of early-onset HCC. Optimal strategies for HCC prevention in these key subroups with chronic HBV warrant further study.

## Introduction

Liver cancer is a leading cause of cancer-related death worldwide, particularly in developing countries [1]. While the incidence of other leading cancers, particularly lung cancer, has declined in recent years due in large part to tobacco control measures, liver cancer rates have continued to increase in many parts of the world [2, 3]. In the United States (US), the incidence of liver cancer has more than doubled in the last three decades [4, 5]. Chronic viral hepatitis due to hepatitis B (HBV) and C (HCV) viruses is thought to account for at least 80% of hepatocellular carcinoma (HCC), the most common form of primary liver cancer [6]. Chronic HBV is estimated to increase the odds of HCC by 50- to 100-fold [7] and contributes overwhelmingly to the disproportionate burden of HCC in Asia and Africa, the world regions with the highest HCC incidence and HBV endemicity [3, 8]. Among chronic HBV-infected patients, there can be significant variability in HCC presentation by race and by geographic region. For example, data from African countries suggest that HCC occurs at younger ages than in other regions of the world, possibly due to greater lifetime exposure to aflatoxin or to HBV genotype [9–11]. These data have lead to US guidelines recommending initiation of HCC surveillance in African-born patients with chronic HBV after the age of 20 [12].

Few studies, however, have examined the risk of HCC in HBV-infected African-born patients who have immigrated to more developed countries. The number of individuals with chronic HBV may be increasing in the US as a result of immigration. From 2004–2008, an estimated 53,800 persons with chronic HBV infection immigrated to the US annually; immigrants now account for 95% of new chronic HBV cases in the US [13]. One population-based study from Minnesota demonstrated that the proportion of HCC attributable to HBV infection rose from 4% to 21% from 2000–2014 [14]. It remains unclear whether HBV-infected patients who immigrate to areas without the environmental risk factors (e.g., dietary iron or aflatoxin) of their native countries continue to have excess risk for HCC and to present at younger ages. More generally, risk factors for HCC have not been well documented among US immigrants with chronic HBV infection, particularly African-born immigrants [15]. Much of the clinical and epidemiologic data on HCC in persons with chronic HBV infection derive from native or immigrant Asian cohorts [12].

We conducted a case-control study to characterize the relative burden of HCC in a diverse population of patients with chronic HBV infection, including African-born patients, seen in a large US urban academic medical setting. Our aim was to describe the spectrum of age at HCC diagnosis and identify demographic and clinical factors associated with HCC in this mostly immigrant population.

## Methods

### Data source and study population

Our data source was a de-identified clinical data repository of all laboratory and clinical diagnoses abstracted from the electronic medical record system of University of Washington Medical Center (UWMC), Harborview Medical Center (HMC) and affiliated outpatient clinics which form a consolidated care network. The UWMC/HMC system provides both primary and specialty care and is the main academic medical center in the greater Seattle region. HMC is the regional safety-net hospital and both centers serve a large community of immigrant patients in King County, Washington. UWMC is a leading referral center for HCC, in part due to its role as the region’s first liver transplantation center. Chronic hepatitis B is identified in both settings as either a known diagnosis on entry to care or through screening as part of routine clinical practice. We selected all patients seen at UWMC or HMC with a diagnosis of chronic hepatitis B and HCC from January 2002 to December 2015. Chronic HBV was defined by International Classification of Diseases, Ninth Revision, Clinical Modification (ICD-9 CM) codes 070.3X, 070.2X and confirmed by chart review. HCC cases were defined by ICD-9 CM 155.0 for hepatocellular carcinoma and confirmed by radiology or clinician documentation on chart review. Non-cancer controls with chronic HBV were matched by age and sex in a 3:1 ratio to patients with HCC. Age in controls was matched to within 3 years of the case’s age to permit this ratio. This research was approved by the Human Subjects Division of the University of Washington.

### Key covariates

We collected demographic data on age, sex and race. We identified immigrant or non-US-born status for black patients and Asians primarily by the presence of non-English (primary spoken) language. For those black patients who had missing or English language, we conducted chart reviews to ascertain African-born status and identified patients as African-born if chart notes indicated country of origin or language. We stratified black patients into African-born and US-born in the race variable for our main analysis because they were the main focus of our inquiry on immigrant status. We did not split Asians by US-born status in the main analysis but did reexamine Asian race stratified as non-US-born versus US-born, using English language as our surrogate for US-born status, as a secondary analysis. HIV coinfection was identified by ICD-9 CM diagnosis codes 042 and V08 and then confirmed by chart review and laboratory results. We defined HCV coinfection as the presence of hepatitis C virus antibody on laboratory testing. We identified diabetes mellitus by ICD-9 CM code 250.X and alcohol use disorder by ICD-9 CM codes 291.x, 303.x and 305.0. Patients were considered to have cirrhosis if they met any of the following criteria: (1) Fibrosis-4 (FIB-4) score greater than 3.6 based on serum aspartate aminotransferase (AST), platelet count, and alanine aminotransferase (ALT) closest to the time of diagnosis [16–18], (2) diagnosis of chronic liver disease and cirrhosis (ICD-9 CM 571.X) or (3) cirrhosis of liver without mention of alcohol (ICD-9 CM 571.5). For additional information on liver disease severity, we collected diagnoses of hepatic complications including ascites (ICD-9 CM 789.5), esophageal varices (ICD-9 CM 456.9, 456.1, 456.2), hepatic encephalopathy (ICD-9 CM 572.2) and spontaneous bacterial peritonitis (ICD-9 CM 567.23).

### Statistical analysis

We compared distributions of categorical variables between cases and controls (or between other strata) by performing Pearson chi squared tests. We compared ages between cases and controls using a two-sample Wilcoxon rank-sum (Mann-Whitney) test. In our primary analysis, we estimated the crude odds ratio (OR) and 95% confidence intervals (CI) for the association between race and the HCC using conditional logistic regression composed of clusters of matched case-control strata. We then calculated adjusted OR, adjusting for several hypothesized risk factors for HCC including HIV coinfection, HCV coinfection, alcohol use disorder, cirrhosis, and diabetes. We also performed a sensitivity analysis without cirrhosis in the model, since HCC can occur without cirrhosis in HBV-infected individuals, and cirrhosis is not always identified in clinical practice [19].

## Results

We identified 296 potential HCC cases who were seen at UWMC or HMC between January 2002 to December 2015. Eighteen cases were excluded after they were determined not to have HCC on chart review, leaving a total of 278 confirmed cases with chronic HBV infection and HCC. A total of 859 controls were identified. After chart review to confirm chronic HBV status, 36 controls who were determined not to have chronic HBV were excluded, leaving a total of 823 controls with chronic HBV but without HCC who were seen between January 2002 and December 2015.

The demographic and clinical characteristics of HCC cases and non-cancer controls are presented in **Table 1**. Over sixty percent of cases and controls were 60 years of age or older, with very few (1.4%) younger than 40. The majority of HCC cases and non-cancer controls were men (78.4% and 78.3%, respectively). As expected, 86.7% of the cases had cirrhosis, compared with only 29% of the non-cancer controls (*P*<0.001). Complications of advanced liver disease including spontaneous bacterial peritonitis, esophageal varices, ascites or hepatic encephalopathy were documented more frequently in cases than controls. There were no statistically significant differences between cases and controls in the percentage of patients with alcohol use diagnoses, diabetes, or HCV coinfection.

**Table 1:** Demographic and clinical characteristics of HCC cases and controls.

	HCC cases n = 278	Controls n = 823	P-value
Age, median (range)	65 (33–93)	64 (30–93)	0.12
Male sex, no. (%)	218 (78)	644 (78)	0.95
Race, no. (%)			<0.001
White	52 (19)	235 (29)*	
Black (overall)	19 (7)	166 (20)	
**Black**: African immigrant	14 (5)	110 (13)	
**Black**: non-immigrant	5 (2)	56 (7)	
**Asian**	199 (72)	394 (48)	
Other	8 (3)	26 (3)	
Alcohol misuse diagnosis, no. (%)	27 (10)	73 (9)	0.67
HIV coinfection^‡^, no. (%)	16 (6)	87 (11)	0.02
Hepatitis C coinfection^‡^, no. (%)	38 (14)	126 (15)	0.51
Diabetes mellitus^‡^, no. (%)	62 (22)	188 (23)	0.85
Cirrhosis^‡^, no. (%)	241 (87)	239 (29)	<0.001
Liver complications^‡^, no. (%)			
Any of the following	103 (37)	79 (10)	<0.001
Ascites	37 (13)	47 (6)	<0.001
Hepatic encephalopathy	37 (13)	26 (3)	<0.001
Esophageal varices	58 (21)	24 (3)	<0.001
Spontaneous bacterial peritonitis	12 (4)	10 (1)	<0.001

*Among 821 controls with non-missing race.

^**‡**^Definitions–Cirrhosis: FIB4 index >3.6 or ICD-9 CM code 571.x. HIV: ICD-9 CM codes 042, V08. Hepatitis C: positive hepatitis C virus antibody. Alcohol misuse: ICD-9 CM codes 291.x, 303.x or 305.0. Diabetes mellitus: ICD-9 CM code 250.x. Liver complications: respective ICD-9 CM codes for these diagnoses

### Race, age and disease severity at HCC diagnosis

Asians were the predominant racial group among patients with HCC, comprising 71.6% (n = 199) of the 278 cases. Median age at HCC diagnosis among Asians was 59 years (range 27–88). Men comprised 75% of those with HCC and cirrhosis was present in 86% of these Asian cases. The countries most commonly represented among the non-English speaking Asian cases were Vietnam, China and Korea.

**Fig 1:**
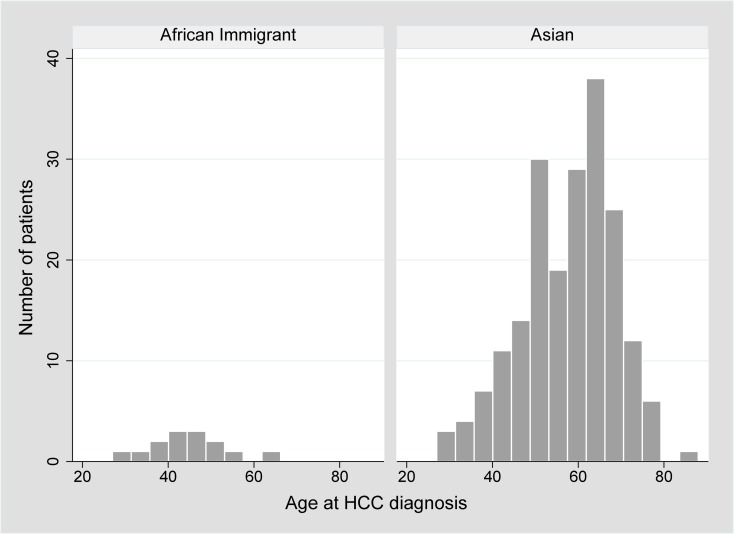
Demonstrates the distribution of age at HCC diagnosis across both African immigrants and Asians. Seventy-one percent (n = 10) of African cases were diagnosed with HCC at an age younger than 50 years compared with 22% (n = 44) of Asian cases (*P*<0.001). Overall there were 71 patients who were diagnosed with HCC at an age younger than 50 years. The majority (62%) were Asian, 14% were African immigrant and 18% white. Eleven (15%) were HIV-coinfected and 77% had cirrhosis.

Black patients represented only 6.8% (n = 19) of all cases of HCC in the 14-year period of the study despite comprising 20% (n = 166) of the controls (*P*<0.001). Five percent (n = 14) of the HCC cases and 13% (n = 110) of controls were African immigrants. Most (86%, n = 12) of the HCC cases were of East African origin (mainly Ethiopia and Somalia), consistent with the demographics of African immigrants in King County, Washington [20]; the remaining two were from West African countries (Chad and Liberia). The median age at HCC diagnosis was 44 years (range 28–65), **Fig 1**. Notably, nearly all (93%) of the African patients were diagnosed with HCC at an age younger than 60 years compared with 52% of Asian cases (*P*<0.001). Most (79%, n = 11) African cases were men and 64% (n = 9) of patients met our critiera for cirrhosis. Of note, 89% (n = 8) of cirrhotic patients with HCC were diagnosed at an age younger than 50 years (median age 42). Among the African immigrant patients with HCC, 7% (n = 1) had HIV co-infection.

### Factors associated with HCC

Asian race was the only race significantly associated with greater odds of HCC in univariate and multivariate analysis (**Table 2**) with an adjusted odds ratio (aOR) of 3.3 (95% CI 1.9–5.5) compared with white patients, after adjusting for diabetes, HIV or HCV coinfection, alcohol misuse and cirrhosis. The aOR for HCC in African immigrants was also elevated but to a lesser extent and did not meet statistical significance (aOR 1.3, 95% CI 0.6–2.9). Black non-immigrant status did not appear to be associated with HCC.

**Table 2:** Factors associated with hepatocellular carcinoma.

	Crude OR	95% CI	P-value	Adjusted OR^‡^	95% CI	P-value
Race (reference: White)						
African-immigrant	0.6	0.3–1.1	0.12	1.3	0.6–2.9	0.58
Black non-immigrant	0.4	0.2–1.1	0.08	0.6	0.2–2.0	0.43
Asian	2.3	1.6–3.3	<0.001	3.3	1.9–5.5	<0.001
Other	1.4	0.6–3.2	0.44	1.9	0.6–5.7	0.27
HIV coinfection	0.5	0.3–0.9	0.02	0.8	0.4–1.8	0.66
HCV coinfection	0.9	0.6–1.3	0.56	0.9	0.6–1.6	0.80
Diabetes	1.0	0.7–1.3	0.77	0.6	0.4–0.9	0.03
Alcohol	1.1	0.7–1.8	0.63	0.9	0.5–1.7	0.71
Cirrhosis	17.3	11.1–27	<0.001	19.7	12.2–31.8	<0.001

OR, odds ratio; CI, confidence interval; HIV, human immunodeficiency virus; HCV, chronic hepatitis C.

^**‡**^ Multivariable conditional logistic regression model that included all of the factors noted in the table. Cases and controls were matched for age and sex.

Cirrhosis had the strongest independent association with HCC overall, crude OR 17.3 (95% CI 11.1–27) and aOR 19.7 (95% CI 12.2–31.8). Diabetes was associated with decreased odds of HCC, aOR 0.6 (95% CI 0.4–0.9) in our primary model but not in our secondary analysis excluding cirrhosis (see below). HCV or HIV co-infections and alcohol use were not associated with increased odds of HCC in our main analysis.

In the secondary multivariable analysis that did not include cirrhosis (S1 Table, Asian race remained associated with HCC with aOR 2.6 (95% CI 1.7–3.9). In contrast to the primary analysis, diabetes was no longer associated with HCC, aOR 1.0 (95% CI 0.7–1.4). Alcohol use disorder was, however, associated with a greater odds of HCC in this model without cirrhosis, aOR 1.6 (95% CI 0.9–2.7, *P* = 0.08).

We also reexamined the multivariable model (including cirrhosis) with Asian race stratified as US-born versus not, using English language as our surrogate for US-born status. Among the 593 Asians, 171 (29%) were noted to be English speakers. Asians, either primary English or non-English speakers, had elevated risk for HCC (see S2 Table).

## Discussion

We conducted a case-control study set in a large academic medical center and public safety-net hospital serving a heterogeneous population of patients with chronic hepatitis B including African immigrants. Among our mostly immigrant cohort, we identified 278 HCC cases over a 14-year span. Our cases were predominantly male, consistent with the prior observation that men carry a 4-fold higher risk of HCC than women [21, 22]. Asian race and cirrhosis were the independent factors most strongly associated with HCC after adjusting for key demographic and clinical factors, with Asians and cirrhotics comprising the majority of our cases. African-born patients comprised the majority of black patients with chronic hepatitis B in our cohort, consistent with the known epidemiology of this infection. They comprised 5% of all HCC cases (and 13% of controls) observed during this time and African immigrant status was not significantly associated with excess HCC risk compared with whites. Africans were however diagnosed, on average, at a younger age than HCC cases in Asians.

Studies have shown that African-born patients, particularly West Africans, are at increased risk for developing HCC at an earlier age than other populations [10, 11, 23–25]. However this finding has not been established in immigrant patients, specifically those with chronic hepatitis B, from these regions. Country of birth rather than race/ethnicity was an independent risk factor for early-onset HCC in one study based on the Surveillance, Epidemiology, and End Results (SEER) cancer registry, in which origin from West Africa or, to a lesser extent, Central/South or East Africa had a strong association with onset of HCC at ages younger than 40 years [9]. However, the SEER study did not capture data on liver disease severity or presence of viral hepatitis which are key determinants of HCC risk. Our study is the first to document this risk of early-onset HCC specifically in African-born immigrants with chronic HBV. The median age at HCC diagnosis in our study (i.e., 44 years) is comparable to those reported in native African cohorts [11]. The mechanism for this earlier presentation remains unclear. Screening for HCC at younger ages for African-born patients is unlikely to account fully for this given the lengthy period of case finding. Longer duration of chronic HBV infection may play a role but would not fully explain why African-born patients had younger median age at diagnosis than Asians, who frequently acquire the infection perinatally. Increased exposure to dietary iron or to aflatoxin (a mycotoxin produced in grains and peanuts that have been stored in warm, moist conditions and a known environmental risk factor for HCC), particularly among African immigrants, have been postulated to potentiate this early risk for HCC [23]. The role of genetic susceptibility to HCC has not been fully explored across multiple races, and most studies of candidate single nucleotide polymorphisms or genome wide association have been conducted primarily in Asians or Caucasians than Africans [26]. Regional differences in viral factors–HBV genotype, delta coinfection and specific genetic mutations–may also contribute to hepatocarcinogenesis [27–29] in ways not yet fully elucidated.

Our findings do underscore the greater odds of HCC in Asian patients overall. This increased risk may be attributable to HBV genotype C, prevalent in and specific to Asia, which has been linked with greater risk of HCC [30]. Greater time with higher HBV viremia may also be a contributing factor, as suggested by one study that compared Chinese and Senegalese patients with HBV infection [31] and other longitudinal cohort studies of patients with chronic HBV that have identified perinatal acquisition as a risk factor for significant fibrosis and HCC [32].

Cirrhosis had the strongest association for HCC in our study, and was present in the majority of chronic HBV patients with HCC regardless of their race/ethnicity. Unlike other causes of HCC where cirrhosis is nearly ubiquitous in HCC, HBV is an oncogenic virus that can, through epigenetic changes in the liver microenvironment, potentiate HCC independent of cirrhosis. However HCC incidence is several orders of magnitude higher in HBV-infected patients with cirrhosis than in those without [30, 33]. Five-year cumulative risk for HCC may be as high as 15% in untreated chronic HBV patients with cirrhosis [33, 34]. While older literature has suggested that African patients may be more likely to present with HCC without cirrhosis [35], our findings suggest that, even in younger African-born patients, cirrhosis remains a key contributor to HCC and that liver disease staging needs to occur even in these younger patients.

Diabetes was not associated with HCC in our univariate analysis but when we adjusted for cirrhosis, diabetes had a reduced odds for HCC. We believe this is likely an artifact of the unexpectedly low prevalence of diabetes in the small subgroup of patients with HCC and no cirrhosis (3 of 37, or 8%) compared with controls without HCC or cirrhosis (80 of 239, 33%) in our cohort. Diabetes and metabolic syndrome have both been shown to be associated with an increased risk of HCC in population-based studies [36, 37]. Additionally, alcohol use disorder was only associated with greater odds of HCC in our multivariable model without cirrhosis, suggesting that its contribution to excess HCC risk may be largely mediated through cirrhosis.

Limitations of this study include the retrospective nature and the inability of a cross-sectional, case-control design to establish causal association or provide an estimate of HCC incidence. The actual incidence rate ratio for HCC in these key subsets of patients with chronic HBV is uncertain and cannot be derived from the present study. We were not able to assess or adjust for variability in HCC screening practices, if they existed, between different subgroups of patients. While differences in screening intervals or practices may have accounted for some of our findings, studies suggest that screening guidelines are infrequently and inconsistently followed in clinical practice across multiple patient groups [38–41] and that the majority of HCC has historically been diagnosed outside of surveillance [14, 42]. Misclassification is possible when relying on clinical diagnostic codes but we were able to confirm the accuracy of diagnoses for chronic HBV infection and HCC by chart review. The use of non-English language as an indicator for non-US-born may only capture a portion of all such individuals, and we may underrepresent HBV-endemic countries like India with this approach. We did not have data on delta coinfection or HBV-specific data such as e antigen status or more importantly HBV DNA levels, which have been shown to be associated with increased risk of HCC [43, 44]. We also did not include HBV treatment, a potentially important modifier of HCC [45], because of our concern for missing data and inadequate capture in our system. We may not have identified all HCC diagnoses in our patient population since some patients could have been diagnosed or have received HCC care outside our system. Our sample of African-born patients with HCC was small, thus our finding no association with African-born status and HCC should be interpreted with caution. This study was conducted in an urban academic medical setting and therefore subject to potential referral and/or selection bias; our findings may also not be generalizable to other populations or settings. Despite these limitations, our study included a large diverse population of patients with chronic HBV infection, including a variety of non-US-born persons, seen over a 14-year timespan and offers a contemporary examination of race/ethnicity as a risk factor for HCC, independent of other key clinical factors.

In summary, Asian race and cirrhosis were the main risk factors and most strongly associated with HCC overall in our study sample. While African immigrants accounted for a small proportion of HBV-associated HCC cases in our cohort and did not appear to have excess risk of HCC overall, they were diagnosed with HCC at younger ages than other racial groups. Further work needs to be done to evaluate and overcome the structural, cultural and patient- and provider-specific barriers that impede the timely diagnosis and optimal care of patients with chronic HBV infection. Non-US-born patients may–due to language, stigma and socioeconomic factors–be particularly vulnerable to delayed entry to appropriate medical care and missed opportunities in the prevention of HCC [46].

## Supporting information

S1 Table Factors associated with hepatocellular carcinoma, multivariable model without cirrhosis. (DOCX)

S2 Table Factors associated with hepatocellular carcinoma, multivariable model with cirrhosis with Asian race stratified. (DOCX)
