# Supplementary material for: Hepatocellular carcinoma among US and non-US-born patients with chronic hepatitis B: Risk factors and age at diagnosis
Source: PLoS One. 2018 Sep 25;13(9):e0204031. doi: 10.1371/journal.pone.0204031 (PMC6155504; doi:10.1371/journal.pone.0204031)
Supplement: S1 Table — (DOCX) [file pone.0204031.s001.docx]

**Supplemental Table 1: Factors Associated with Hepatocellular Carcinoma, Multivariable Model without Cirrhosis**

|  | Adjusted OR^‡^ | 95% CI | P-value |
| --- | --- | --- | --- |
| Race (reference: White)  African-immigrant  Black non-immigrant  Asian  Other | 0.7  0.4  2.6  1.4 | 0.3-1.3  0.2-1.1  1.7-3.9  0.6-3.2 | 0.23  0.09  <0.001  0.48 |
| HIV coinfection | 0.8 | 0.4-1.5 | 0.43 |
| HCV coinfection | 1.2 | 0.8-1.8 | 0.48 |
| Diabetes | 1.0 | 0.7-1.4 | 0.86 |
| Alcohol | 1.6 | 0.9-2.7 | 0.08 |

OR, odds ratio; CI, confidence interval; HIV, human immunodeficiency virus; HCV, chronic hepatitis C. **^‡^** Multivariable conditional logistic regression model that included all of the factors noted in the table. Cases and controls were matched for age and sex.
