# Supplementary material for: Hepatocellular carcinoma among US and non-US-born patients with chronic hepatitis B: Risk factors and age at diagnosis
Source: PLoS One. 2018 Sep 25;13(9):e0204031. doi: 10.1371/journal.pone.0204031 (PMC6155504; doi:10.1371/journal.pone.0204031)
Supplement: S2 Table — (DOCX) [file pone.0204031.s002.docx]

**Supplemental Table 2: Factors Associated with Hepatocellular Carcinoma, Multivariable Model with Cirrhosis with Asian race stratified**

|  | Adjusted OR^‡^ | 95% CI | P-value |
| --- | --- | --- | --- |
| Race (reference: White)  African-immigrant  Black non-immigrant  Asian* (non-English)  Asian* (English)  Other | 1.3  0.6  3.0  4.0  1.9 | 0.6-3.0  0.2-2.0  1.8-5.2  2.1-7.6  0.6-5.9 | 0.56  0.44  <0.001  <0.001  0.25 |
| HIV coinfection | 0.8 | 0.4-1.8 | 0.64 |
| HCV coinfection | 0.9 | 0.6-1.6 | 0.82 |
| Diabetes | 0.6 | 0.4-1.0 | 0.03 |
| Alcohol | 0.9 | 0.5-1.7 | 0.68 |
| Cirrhosis | 20.0 | 12.3-32.4 | <0.001 |

OR, odds ratio; CI, confidence interval; HIV, human immunodeficiency virus; HCV, chronic hepatitis C. **^‡^** Multivariable conditional logistic regression model that included all of the factors noted in the table. Cases and controls were matched for age and sex. *Asian race stratified by primary spoken language (English and non-English).
